# Incidence, genetic diversity, and antimicrobial resistance profiles of *Vibrio parahaemolyticus* in seafood in Bangkok and eastern Thailand

**DOI:** 10.7717/peerj.15283

**Published:** 2023-05-11

**Authors:** Chartchai Changsen, Somsak Likhitrattanapisal, Kamonwan Lunha, Wiyada Chumpol, Surasak Jiemsup, Anuphap Prachumwat, Darin Kongkasuriyachai, Supawadee Ingsriswang, Soraya Chaturongakul, Aekarin Lamalee, Suganya Yongkiettrakul, Sureemas Buates

**Affiliations:** 1 Department of Microbiology, Faculty of Science, Mahidol University, Bangkok, Thailand; 2 National Center for Genetic Engineering and Biotechnology (BIOTEC), National Science and Technology Development Agency (NSTDA), Pathum Thani, Thailand; 3 AQHT, AAQG, National Center for Genetic Engineering and Biotechnology (BIOTEC), National Science and Technology Development Agency (NSTDA), Bangkok, Thailand; 4 CENTEX SHRIMP, Faculty of Science, Mahidol University, Bangkok, Thailand; 5 Molecular Medical Biosciences Cluster, Institute of Molecular Biosciences, Mahidol University, Nakhon Pathom, Thailand

**Keywords:** *V. parahaemolyticus*, Seafood, Antimicrobial resistance, Genetic diversity, Multilocus sequence typing, Thailand

## Abstract

**Background:**

Emergence of *Vibrio parahaemolyticus* pandemic strain O3:K6 was first documented in 1996. Since then it has been accounted for large outbreaks of diarrhea globally. In Thailand, prior studies on pandemic and non-pandemic *V. parahaemolyticus* had mostly been done in the south. The incidence and molecular characterization of pandemic and non-pandemic strains in other parts of Thailand have not been fully characterized. This study examined the incidence of *V. parahaemolyticus* in seafood samples purchased in Bangkok and collected in eastern Thailand and characterized *V. parahaemolyticus* isolates. Potential virulence genes, VPaI-7, T3SS2, and biofilm were examined. Antimicrobial resistance (AMR) profiles and AMR genes (ARGs) were determined.

**Methods:**

*V. parahaemolyticus* was isolated from 190 marketed and farmed seafood samples by a culture method and confirmed by polymerase chain reaction (PCR). The incidence of pandemic and non-pandemic *V. parahaemolyticus* and VPaI-7, T3SS2, and biofilm genes was examined by PCR. AMR profiles were verified by a broth microdilution technique. The presence of ARGs was verified by genome analysis. *V. parahaemolyticus* characterization was done by multilocus sequence typing (MLST). A phylogenomic tree was built from nucleotide sequences by UBCG2.0 and RAxML softwares.

**Results:**

All 50 *V. parahaemolyticus* isolates including 21 pathogenic and 29 non-pathogenic strains from 190 samples had the *toxRS/*old sequence, indicating non-pandemic strains. All isolates had biofilm genes (VP0950, VP0952, and VP0962). None carried T3SS2 genes (VP1346 and VP1367), while VPaI-7 gene (VP1321) was seen in two isolates. Antimicrobial susceptibility profiles obtained from 36 *V. parahaemolyticus* isolates revealed high frequency of resistance to colistin (100%, 36/36) and ampicillin (83%, 30/36), but susceptibility to amoxicillin/clavulanic acid and piperacillin/tazobactam (100%, 36/36). Multidrug resistance (MDR) was seen in 11 isolates (31%, 11/36). Genome analysis revealed ARGs including *blaCARB* (100%, 36/36), *tet(34)* (83%, 30/36), *tet(35)* (42%, 15/36), *qnrC* (6%, 2/36), *dfrA6* (3%, 1/36), and *blaCTX-M-55* (3%, 1/36). Phylogenomic and MLST analyses classified 36 *V. parahaemolyticus* isolates into 5 clades, with 12 known and 13 novel sequence types (STs), suggesting high genetic variation among the isolates.

**Conclusions:**

Although none *V. parahaemolyticus* strains isolated from seafood samples purchased in Bangkok and collected in eastern Thailand were pandemic strains, around one third of isolates were MDR *V. parahaemolyticus* strains. The presence of resistance genes of the first-line antibiotics for *V. parahaemolyticus* infection raises a major concern for clinical treatment outcome since these resistance genes could be highly expressed under suitable circumstances.

## Introduction

Seafood is generally a good source of high-quality proteins, essential amino acids, and healthy fats for a lower calorie intake per portion compared to other animal meats. Due to the increase in global consumption of seafood products, oversight of seafood quality is a priority to prohibit contamination from seafood-borne pathogens (FAO, 2020; Choudhury et al., 2022). *Vibrio parahaemolyticus* is a Gram-negative bacterium commonly found in seawater, seafood (particularly oysters), and aquatic products (Nakaguchi, 2013; Odeyemi, 2016). It is a leading cause of human gastroenteritis following consumption of raw or improperly cooked contaminated seafood (Martinez-Urtaza & Baker-Austin, 2020). Pathogenicity of *V. parahaemolyticus* infection has been attributed to the expression of virulent determinant genes including *tdh* (thermostable direct hemolysin, TDH), *trh* (TDH-related hemolysin, TRH), pathogenicity islands (PAIs), type III secretion system (T3SS1 and T3SS2), and biofilm formation genes (Raghunath, 2015; Klein, Pipes & Lovell, 2018; Li et al., 2019; Sharan et al., 2022). *V. parahaemolyticus* strains are classified into pathogenic, non-pathogenic, pandemic, and non-pandemic strains (Okura et al., 2003; Meador et al., 2007; Chao et al., 2009; Lopez-Joven et al., 2015). Pathogenic strains harbor *tdh* and/or *trh* genes while non-pathogenic strains lack both *tdh* and *trh* genes. Pandemic strains harbor both *tdh* gene and *toxRS/*new gene (the pandemic marker gene containing the unique base changes in the regulatory gene, *toxRS*). Non-pandemic strains lack both *tdh* and *toxRS/*new genes genes (Okura et al., 2003; Meador et al., 2007; Chao et al., 2009; Lopez-Joven et al., 2015).

The first pandemic strain of *V. parahaemolyticus* O3:K6 was documented in February 1996 in Kolkata, India (Okuda et al., 1997). Infections caused by the O3:K6 strain and related serovariants, collectively called pandemic strains, were responsible for several large gastroenteritis outbreaks in humans (Nair et al., 2007). *V. parahaemolyticus* new/pandemic O3:K6 strains isolated since 1996 produced only TDH while the old O3:K6 strains isolated before 1996 produced only TRH (Okuda et al., 1997; Honda, Ni & Miwatani, 1988). Several studies have detected pandemic *V. parahaemolyticus* serovariants not only in clinical samples (Li et al., 2014; Pazhani et al., 2014; Ueno et al., 2016), but also in seafood and other environmental samples (Arakawa et al., 1999; Deepanjali et al., 2005; Quilici et al., 2005; Caburlotto et al., 2010; Haley et al., 2014; Jingjit et al., 2021), indicating that the pandemic strains have established ecological niches around the world. Although *tdh* and *trh* are associated with pathogenic strains, several studies have reported about 10% of clinical strains do not contain *tdh* and *trh* suggesting that pathogenicity could be caused by additional virulence factor(s) (Jones et al., 2012; Li et al., 2014; Pazhani et al., 2014).

The pathogenic potentials of *V. parahaemolyticus* are also associated with increased resistance to antimicrobials because of excessive use and misuse of antimicrobials in humans, agriculture, and aquaculture systems (Mazel & Davies, 1999; Cabello, 2006; Igbinosa, 2016). In recent reports, *V. parahaemolyticus* strains isolated from seafood, clinical, and environmental samples were highly resistant to multiple antibiotics including amoxicillin, ampicillin, ciprofloxacin, cefazolin, ceftazidime, cefotaxime, cefuroxime sodium, colistin, gentamicin, penicillin, spectinomycin, tetracycline and doxycycline (Tan et al., 2020; Ashrafudoulla et al., 2021; Dutta et al., 2021). Among antibiotic-resistant profiles, tetracycline, doxycycline, a 3^rd^-generation cephalosporin, and quinolone including ciprofloxacin are recommended for the treatment of severe or prolonged *V. parahaemolyticus* infection (Ashrafudoulla et al., 2021; Dutta et al., 2021; Rezny & Evans, 2022*)*. Several studies demonstrated that seafood is a potential reservoir for the dissemination of multidrug resistant (MDR) *V. parahaemolyticus* (Letchumanan et al., 2015; Jeamsripong, Khant & Chuanchuen, 2020; Li et al., 2020*)*.

In Thailand, *V. parahaemolyticus* is the major causative agent of human gastroenteritis, occurring in about 50–60% of gastroenteritis cases (DDC, 2020). Incidence of human gastroenteritis in Thailand has increased every year and has been linked to consumption and mishandled of seafood contaminated with *V. parahaemolyticus* (DDC, 2020). The first report of the O3:K6 pandemic strain was obtained from patients at Hat Yai Hospital located in southern Thailand in 1988 (Matsumoto et al., 2000). Since then the studies of pandemic and non-pandemic *V. parahaemolytics* strains have been mainly conducted in southern parts of the country (Vuddhakul et al., 2000; Laohaprertthisan et al., 2003; Vuddhakul et al., 2006; Wootipoom et al., 2007; Bhoopong et al., 2007; Thongjun et al., 2013; Han et al., 2016; Jingjit et al., 2021). Molecular fingerprinting methods have suggested possible epidemiological linkage between the clinical and the environmental strains in southern Thailand (Vuddhakul et al., 2006). Very limited information on occurrence and molecular characterization of pandemic and non-pandemic strains is available in other areas of Thailand with high concentration of seafood sources. This study aimed to investigate the occurrence of *V. parahaemolyticus* pandemic and non-pandemic strains, the characterization of *V. parahaemolyticus* isolates, the presence of pathogenic potential genes including VPaI-7, T3SS2 and biofilm formation genes, and AMR profiles of *V. parahaemolyticus* isolated from raw seafood in Bangkok and eastern Thailand (highly populated areas). These findings will add to the body of knowledge and surveillance information of *V. parahaemolyticus* that would be useful for relevant stakeholders to make informed-decision in the aquaculture management, food safety, and public health strategies.

## Materials and Methods

### Sample collection and *V. parahaemolyticus* isolation

Sample size estimation was based on the assumption of previous prevalence of 59% of *V. parahaemolyticus* contamination in seafood collected in Bangkok, Thailand (Atwill & Jeamsripong, 2021). The formula (*n* = *Z*^2^*P*(1−*P*)/*d*^*2*^), 95% confidence level and 7% margin of error were used in this study to obtain the representative sample with a less expensive survey (Daniel, 1999; Pourhoseingholi, Vahedi & Rahimzadeh, 2013). In this formula, *n* is the sample size, *Z* is the statistic for a level of confidence, *P* is expected prevalence, and *d* is precision or margin of error. The required samples were 190 samples which were collected from different sources, time, and locations. Of 190 samples, 143 and 31 samples were purchased in Bangkok, Thailand in 2018 and 2021 to 2022, respectively and 16 samples were collected from different shrimp farms in eastern Thailand in 2013. Among 174 of samples purchased in Bangkok, Thailand, 84 and 90 samples were purchased from fresh markets and supermarkets, respectively. Of 90 samples purchased from supermarkets, 40 samples were frozen seafood. One hundred and ninety samples comprised of crabs (*n* = 20; blue swimming crabs, red swimming crabs, and mud crabs), fish (*n* = 50; groupers, mackerels, ornate threadfin bream, and giant seaperch), mollusc shellfish (*n* = 50; blood cockles, green mussels, oysters, short-necked clams, and spiral babylon snails), shrimp (*n* = 50; giant tiger prawns and Pacific white shrimp) and squids (*n* = 20; splendid squids, Dollfus’ octopuses, and giant squid tentacle).

For sample purchasing, markets were chosen based on geography to ensure complete coverage of Bangkok and each market was visited only once. After purchasing from markets or collecting from farms, samples were individually packed in a sterilized plastic bag and transported on ice to the laboratory and processed within 2 h. For *V. parahaemolyticus* isolation from marketed and farmed samples, a 2.5-g portion of seafood sample was aseptically transferred into a stomacher bag containing 22.5 mL of tryptic soy broth (TSB) supplemented with 2% NaCl, mixed thoroughly by hand for 1 min and kept at room temperature for 30 min. Thereafter, the sample was removed from the broth which was incubated at 37 °C for 16 h before culturing on selective media of thiosulfate citrate bile salts sucrose agar (TCBSA, Difco Laboratories, Franklin Lakes, NJ, USA). *V. parahaemolyticus* colonies were opaque and blue-green color with 2–3 mm in diameter on the TCBS agar plates. *V. parahaemolyticus* colonies were further confirmed using CHROMagar™ Vibrio (CHROMagar, Paris, France) of which the positive colonies gave mauve color and were collected for further characterization (Ahmmed et al., 2019).

### Polymerase chain reaction (PCR)

The isolates obtained from CHROMagar™ Vibrio were further confirmed for *V. parahaemolyticus* by PCR using species-specific PCR primers targeting *toxR* gene (Kim et al., 1999). To identify the pandemic O3:K6 strain and its serovariants, PCR primers targeting *tdh* and *toxRS/*new genes were used (Tada et al., 1992; Matsumoto et al., 2000). PCR primers targeting *toxRS/*old were used for determining the O3:K6 strains isolated before 1996 (Okura et al., 2003). PCR primers targeting one VPaI-7 open reading frame (ORF), two TSSS2 genes, and three biofilm genes, were used to identify genes involved in pathogenesis (Chao et al., 2009; Chao et al., 2010). PCR primers and conditions used in this study were summarized in Table 1. Briefly, the PCR reaction was performed in a 25-μL reaction volume, containing 12.5 μL GoTaq® Green Master Mix solution (Promega, Madison, WI, USA), 2 μL of DNA template (50 to 70 ng) and milli-Q water for adjusting final volume up to 25 μL. Gene-specific primer sets were added to corresponding PCR reactions: 0.1 μM of each primer for *tdh* gene; 0.6 μM of each primer for three biofilm genes; 0.8 μM of each primer for *toxR*, *toxRS*/old, and *toxRS*/new genes; and 1 μM of each primer for VPaI-7 and T3SS2 genes. PCR amplifications were performed in triplicate for each sample. PCR products were analyzed by electrophoresis at 75 V for 40 min in 1.5% agarose (Vivantis Technologies, Malaysia) with a 1X TAE (Tris-Acetate + EDTA) buffer. Agarose gels were stained in SYBRTM Safe DNA Gel Stain (Thermo Fisher Scientific, Waltham, MA, USA). Amplicon bands were observed under UV light.

**Table 1:** PCR primers and conditions used in this study.

Primer name	Primer sequence (5′ to 3′)	Target gene	Amplicon size (bp)	Reference	PCR condition
*toxR*-F *toxR*-R	GTCTTCTGACGCAATCGTTG ATACGAGTGGTTGCTGTCATG	*toxR*	368	Kim et al. (1999)	95 °C-30 s; 63 °C-30 s; 72 °C-30 s
					
*toxRS*/old-F *toxRS*/old-R	TAATGAGGTAGAAACG ACGTAACGGGCCTACG	*toxRS* of the old O3:K6 clone	651	Matsumoto et al. (2000)	96 °C-1 min; 47.1 °C-2 min; 72 °C-3 min
					
*toxRS*/new-F *toxRS*/new-R	TAATGAGGTAGAAACA ACGTAACGGGCCTACA	*toxRS* of the new O3:K6 clone	651	Matsumoto et al. (2000)	94 °C-30 s; 52.3 °C-30 s; 72 °C-1 min
					
*tdh*-F *tdh*-R	CCACTACCACTCTCATATGC GGTACTAAATGGCTGACATC	*tdh*	251	Tada et al. (1992)	94 °C-1 min; 55 °C-1 min; 72 °C-1 min
					
VP1321-F VP1321-R	CCTTGGAAGACAAATGTGGAT ATGGCTTACCAATGTCAAACTAT	VPaI-7	261	Chao et al. (2009)	94 °C-40 s; 54.3 °C-40 s; 72 °C-30 s
					
VP1346-F VP1346-R	TACCATCAGAGGATACAACC ACAATGAGAACATCAAACA	VPaI-7 (T3SS2)	262	Chao et al. (2009)	94 °C-40 s; 51.2 °C-40 s; 72 °C-30 s
					
VP1367-F VP1367-R	CTATGGCGTGCTGGTAGAC TCACTCGTAAGATGTTGGG	VPaI-7 (T3SS2)	209	Chao et al. (2009)	94 °C-40 s; 56.6 °C-40 s; 72 °C-30 s
					
VP0950-F VP0950-R	GCCAAACTTCTCAAACAACA ATGAAACGCAATTTACCATC	Biofilm	298	Chao et al. (2010)	94 °C-55 s; 50 °C-50 s; 72 °C-2 min
					
VP0952-F VP0952-R	TATGATGGTGTTTGGTGC TGTTTTTCTGAGCGTTTC	Biofilm	276	Chao et al. (2010)	94 °C-55 s; 50 °C-50 s; 72 °C-2 min
					
VP0962-F VP0962-R	GACCAAGACCCAGTGAGA GGTAAAGCCAGCAAAGTT	Biofilm	358	Chao et al. (2010)	94 °C-55 s; 50 °C-50 s; 72 °C-2 min

### Antimicrobial susceptibility testing

The minimum inhibitory concentrations (MICs), the lowest drug concentration inhibiting visible growth, of different antimicrobial drugs were determined by the broth microdilution technique utilizing a semi-automatic procedure (Sensititre, Trek Diagnostic Systems Ltd., West Sussex, UK) according to Clinical and Laboratory Standards Institute (CLSI) recommendations (CLSI, 2021). Two sets of dehydrated 96-well microtiter plates, including THAN2F and CMV4AGNF were used. In all, 27 antibiotics of different drug classes and action mechanisms were tested in varied concentration as summarized in Table S1. Most of the tested antimicrobial agents in this study are recommended by Centers for Disease Control and Prevention (CDC) for the therapeutics of *Vibrio* spp. infections including fluoroquinolones (ciprofloxacin and levofloxacin), 3^rd^-generation cephalosporins (cefotaxime, ceftazidime, and ceftriaxone), aminoglycosides (gentamicin and amikacin), tetracycline, folate synthesis inhibitors (trimethoprim/sulfamethoxazole) (Daniels & Shafaie, 2000; Shaw et al., 2014). The MIC analysis conditions were done as specified by manufacturer’s guidelines with slight adaptation. Briefly, isolates were cultured overnight on tryptic soy agar (TSA) with 2% NaCl at 37 °C in 5% CO_2_ incubator (Ahmmed et al., 2019). Selected colonies were suspended in Sensititre cation-adjusted Mueller-Hinton broth (CAMHBT) and adjusted to a 0.5 McFarland standard. Thereafter, a 10-μL aliquot of suspension was transferred into a tube of CAMHBT to get an inoculum density of 5 × 10^5^ CFU/mL. THAN2F and CMV4AGNF panels were reconstituted by adding 50 μL/well and were enclosed with an adhesive seal and incubated at 35 ± 2 °C in Sensititre ARIS^TM^ 2X for 20–24 h. The MIC value was determined automatically on the Sensititre ARIS^TM^ 2X and visualized using a manual viewbox in accordance with instructions in Sensititre SWIN software. *Escherichia coli* ATCC 25922 was used as an antimicrobial-susceptible control strain. Results were interpreted according to CLSI, National Antimicrobial Resistance Monitoring System (NARMS), and European Committee on Antimicrobial Susceptibility Testing (EUCAST) breakpoints (CLSI, 2021; NARMS, 2021; EUCAST, 2022). Strains were classified as “non-susceptible” when they were resistant to at least one antimicrobial. Multidrug resistance (MDR) was defined as non-susceptible to at least one agent in three or more antimicrobial classes according to the definition proposed for other bacterial groups (Magiorakos et al., 2012). The Multiple Antibiotic Resistance (MAR) index was determined for each isolate using the formula MAR = a/b, where “a” is the number of antibiotics to which the test isolate is resistant, and “b” is the total number of antibiotics tested. MAR index values greater than 0.2 indicated that the isolates were obtained from a high-risk source of contamination where antibiotics are often used (Krumperman, 1983).

### Whole-genome sequencing (WGS), genome assembly and annotation

To determine the presence of antimicrobial resistance gene (ARG), a pure culture of *V. parahaemolyticus* was grown overnight on TSA with 2% NaCl at 37 °C in 5% CO_2_. Subsequently, colonies were selected and suspended in 5 mL TSB with 2% NaCl. The broth culture was incubated at 37 °C in 5% CO_2_ for 16 h. Thereafter, bacterial cells were collected by centrifugation and used for genomic DNA (gDNA) preparation. *V. parahaemolyticus* gDNA was extracted with phenol-chloroform DNA extraction (Green & Sambrook, 2017). The purity and quantity of gDNA was determined by measuring OD_260/280_ and OD_260/230_ values with a NanoDrop spectrophotometer (Thermo Fisher Scientific, Waltham, MA, USA). The integrity of intact gDNA was verified with agarose gel electrophoresis. Whole-genome sequencing analysis of 36 high quality gDNA samples with OD_260_/OD_280_ of ≥1.8 and OD_260_/OD_230_ of ≥2.0 were performed using Illumina® DNA sequencing with HiSeq 2X150 paired-end (PE) configuration (serviced by GeneWiz Inc., Chelmsford, MA, USA). Raw sequencing reads were trimmed prior to genome assembly using the JGI bbduk tool (k = 27, ktrim = 1, hdist = 1, minlength = 50). The genome assembly and scaffolding were performed using SPAdes version 3.15 (Prjibelski et al., 2020). The genome annotation was performed using Prokka version 1.13 (Seemann, 2014). *V. parahaemolyticus* RIMD 2210633 (accession no. GCA_000196095.1) was used as the reference genome. Sequence data of 36 *V*. *parahaemolyticus* genomes were deposited in DDBJ/EMBL/GenBank. The GenBank accession numbers are shown in Table S2.

### Detection of antimicrobial resistance genes (ARGs)

To detect ARGs in each annotated genome, nucleotide sequences of CDS genes of each annotated genomes were analyzed using ResFinder version 4.1 with minimum sequence alignment coverage = 0.6 and minimum threshold for sequence identity = 0.8 (Florensa et al., 2022).

### Multi-locus sequence typing (MLST) analysis

Molecular typing using MLST was done with 7 conserved housekeeping genes *dnaE*, *gyrB*, *recA*, *dtdS*, *pntA*, *pyrC*, and *tnaA*. Briefly, ORF and scaffold sequences of each isolate were BLASTN searched against the *V. parahaemolyticus* typing allele sequence database downloaded from PubMLST.org (2022-05-09) (Jolley, Bray & Maiden, 2018) to obtain sequence types (STs). The genome sequences of new ST profiles identified in this study were submitted to PubMLST.org databases.

### Phylogenomic tree construction

A maximum-likelihood phylogenomic tree was inferred using UBCG2.0 (version Feb, 2021) (Kim et al., 2021) and RAxML (version 8.2.12) (Stamatakis, 2014) softwares with a default parameter setting, a GTR + CAT substitution model on a nucleotide alignment of 81 bacterial universal core genes from 36 *V. parahaemolyticus* genomes. These universal core genes previously identified by UBCG2 method as single-copy core genes covering 3,508 species of 43 bacterial phyla are suitable for phylogenomic tree analyses (Kim et al., 2021). The phylogenomic tree from the maximum likelihood analysis was visualized with MEGA X software.

## Results

### Detection of *V. parahaemolyticus* potential virulence genes

Among 174 samples purchased from fresh markets and supermarkets, 34 *V. parahaemolyticus* were isolated. A total 50 *V. parahaemolyticus* isolates, 34 isolates from markets and 16 isolates from shrimp farms, were used in this study. Of 50 isolates, 21 and 29 were pathogenic and non-pathogenic strains, respectively (A. Lamalee, 2023, unpublished data) (Table S3). All 50 *V. parahaemolyticus* isolates exhibited positive PCR amplification to *toxR*, *toxRS*/old (a non-pandemic gene marker), and biofilm formation genes (VP0950, VP0952, and VP0962) (Table 2). None of 50 isolates showed positive PCR amplification for both pandemic gene markers (both *tdh* positive and *toxRS*/new positive) and for two T3SS2 genes (VP1346 and VP1367) (Table 2). VPaI-7 (VP1321 ORF) was detected in two isolates (F2CK02 and F3CK01) accounting for 4% (2/50) (Tables 2 and S3). Altogether, the results indicated that all isolates were non-pandemic strains. Based on the presence or absence of genetic markers, 50 *V. parahaemolyticus* isolates could be classified as follows: 21 were non-pandemic and pathogenic strains (*tdh^−^* or *toxRS*/new^−^; *toxRS/*old^+^; *tdh*^+^ or *trh* ^+^) of which two isolates carried VPaI-7 genes, and 29 were non-pandemic and non-pathogenic strains (*tdh*^−^ or *toxRS*/new^−^; *toxRS/*old^+^; *tdh*^−^ and *trh*^−^).

**Table 2:** Distribution of pandemic and non-pandemic strains, and potential pathogenicity genes of 50 *Vibrio parahaemolyticus* isolates.

No. *V. parahae-* *molyticus* isolate	Number positive								
	*toxR*	^1^Non-pandemic	^2^Pandemic	VPaI-7 VP1321	VPaI-7 (T3SS2)		Biofilm		
					VP1346	VP1367	VP0950	VP0952	VP0962
50	50	50	0	2	0	0	50	50	50

**Notes:**

1 Determined by the presence of *toxRS/*old genes.

2 Determined by the presence of both *tdh* and *toxRS/*new.

### Antimicrobial susceptibilities

Of 50 *V. parahaemolyticus* isolates, 14 isolates were not examined for antimicrobial susceptibilities due to poor recovery from glycerol stocks. Thus, antimicrobial susceptibility tests were performed on 36 isolates against 27 agents from five antimicrobial categories (Table S1). Based on the results, the susceptibility rate of 36 *V. parahaemolyticus* strains was 100% (36/36) to fluoroquinolones, carbapenems, amikacin, gentamicin, netilmicin, tetracyclines chloramphenicol, azithromycin, amoxicillin/clavulanic acid, ampicillin/sulbactam, piperacillin/tazobactam, and trimethoprim/sulfamethoxazole (Tables 3 and S4). The susceptible rate was 97% (35/36) to cefoxitin, cefotaxime, ceftazidime, ceftriaxone, and cefepime, 92% (33/36) to sulfisoxazole, and 83% (30/36) to streptomycin. A high number of intermediate susceptibility was observed for cefuroxime (81%, 29/36). The low resistance rate was observed for cefuroxime and sulfisoxazole (8%, 3/36) and for cefotaxime, ceftazidime, ceftriaxone, and cefepime (3%, 1/36). In contrast, high resistance pattern was observed for ampicillin (83%, 30/36) and colistin (100%, 36/36) (Tables 3 and S4).

**Table 3:** Minimum inhibitory concentration (MIC) value, MIC_50_ and MIC_90_ values, and resistance rates of 36 *Vibrio parahaemolyticus* isolates.

Antibiotic drugs	MIC breakpoints, µg/mL			MIC values (µg/mL)^a^																MIC_50_	MIC_90_	S (%)	I (%)	R (%)	MIC ranges
	S	I	R	0.015	0.03	0.06	0.12	0.25	0.50	1	2	4	8	16	32	64	128	256	512						
Amikacin	≤16	32	≥64										36	0|	0∣					≤8	≤8	100.0	0.0	0.0	≤8
Amoxicillin-clavulanic acid	≤8/4	16/8	≥32/16							1	31	4	0|	0∣	0					2	4	100.0	0.0	0.0	≤1–4
Ampicillin	≤8	16	≥32							0	0	0	0|	6∣	18	12				32	>32	0.0	16.7	83.3	16–>32
Ampicillin-sulbactam	≤8/4	16/8	≥32/16									35	1|	0∣						≤4	≤4	100.0	0.0	0.0	≤4–8
Azithromycin	≤4	–	>4					26	10	0	0∣	0	0	0	0					≤0.25	0.5	100.0	–	0.0	≤0.25–0.5
Cefepime	≤2	4-8	≥16							35	0|	0	0∣	0	1					≤1	≤1	97.2	0.0	2.8	≤1–>32
Cefotaxime	≤1	2	≥4							35|	0∣	0	0	0	1					≤1	≤1	97.2	0.0	2.8	≤1–>32
Cefoxitin	≤8	16	≥32						0	0	0	7	28|	1∣	0					8	8	97.2	2.8	0.0	4–16
Ceftazidime	≤4	8	≥16							35	0	0|	0∣	1	0					≤1	≤1	97.2	0.0	2.8	≤1–16
Ceftriaxone	≤1	2	≥4					35	0	0|	0∣	0	0	0	0	1				≤0.25	≤0.25	97.2	0.0	2.8	≤0.25–>64
Cefuroxime (sodium)	≤8	16	≥32										4|	29∣	3					16	16	11.1	80.6	8.3	≤8–>16
Chloramphenicol	≤8	16	≥32								36	0	0|	0∣	0					≤2	≤2	100.0	0.0	0.0	≤2
Ciprofloxacin	≤1	2	≥4			4	24	8	0	0|	0∣	0								0.12	0.25	100.0	0.0	0.0	0.06–0.25
Colistin	≤2	–	>2							0∣	0	2	2	32						>8	>8	0.0	–	100.0	4–>8
Doripenem	≤1	2	≥4						36	0|	0∣	0	0	0						≤0.5	≤0.5	100.0	0.0	0.0	≤0.5
Ertapenem	≤0.5	1	≥2						36|	0∣	0	0								≤0.5	≤0.5	100.0	0.0	0.0	≤0.5
Gentamicin	≤4	8	≥16					0	0	7	26	3|	0∣	0						2	2	100.0	0.0	0.0	1–4
Imipenem	≤1	2	≥4						36	0|	0∣	0	0	0						≤0.5	≤0.5	100.0	0.0	0.0	≤0.5
Levofloxacin	≤2	4	≥8			4	28	4	0	0	0|	0∣	0							0.12	0.25	100.0	0.0	0.0	≤0.06–0.25
Meropenem	≤1	2	≥4			36	0	0	0	0|	0∣	0	0	0						≤0.06	≤0.06	100.0	0.0	0.0	≤0.06
Nalidixic acid	≤16	–	≥32						25	10	1	0	0	0|	0∣					≤0.5	1	100.0	–	0.0	≤0.5–2
Netilmicin	≤8	16	≥32										36|	0∣						≤8	≤8	100.0	0.0	0.0	≤8
Piperacillin-tazobactam	≤16/4	32/4-64/4	≥128/4										36	0|	0	0∣				≤8	≤8	100.0	0.0	0.0	≤8
Streptomycin	≤16	–	≥32								0	0	0	30∣	6	0				16	32	83.3	–	16.7	16–32
Sulfisoxazole	≤256	–	≥512											2	9	15	5	2∣	3	64	256	91.7	–	8.3	≤16–>256
Tetracycline	≤4	8	≥16									36|	0∣	0	0					≤4	≤4	100.0	0.0	0.0	≤4
Trimethoprim-sulfamethoxazole	≤2/38	–	≥4/76				35	0	1	0	0∣	0								≤0.12	≤0.12	100.0	–	0.0	≤0.12–0.5

**Note:**

a White cells indicate the tested range. Thin and thick vertical lines respectively describe the susceptible and resistant clinical breakpoints recommended by the CLSI (2021) for most of the antibiotic drugs, NARMS (2021) for streptomycin, and EUCAST (2022) for azithromycin and colistin. MICs are interpreted as susceptible (S), intermediate (I), and resistant (R). MIC_50_ is the MIC which inhibits 50% of the isolates tested; MIC_90_ is the MIC which inhibits 90% of the isolates tested.

AMR patterns of 36 *V. parahaemolyticus* isolates could be classified into six patterns in which pattern No. 2 (AMP/COL) was predominant (53%, 19/36) (Table 4). MDR *V. parahaemolyticus* was observed in 11 isolates (31%,11/36) and all of them exhibited resistance to ampicillin and colistin. Interestingly, one MDR isolate, VP42 isolated from Pacific white shrimp, exhibited additional resistance to cefuroxime, cefotaxime, ceftazidime, ceftriaxone, and cefepime. All 36 *V. parahaemolyticus* isolates exhibited MAR index range of 0.04 to 0.3, indicating that *V. parahaemolyticus* isolates were resistant to 1–8 types of tested antibiotics (Table S5). VP42 showed resistant to 8/27 antimicrobial agents, which corresponded to the highest MAR index (0.3). The MAR index value greater than 0.2 suggested that VP42 was isolated from a high-risk source of antimicrobial contamination (Krumperman, 1983).

**Table 4:** Antimicrobial resistance (AMR) patterns of 36 *Vibrio parahaemolyticus* isolates.

Resistance pattern	Number of *V. parahaemolyticus* isolate	Percentage (%)
1. COL	6	16.7
2. AMP/COL	19	52.8
3. AMP/COL/S	6	16.7
4. AMP/COL/SIX	2	5.6
5. AMP/COL/FUR	2	5.6
6. AMP/CPM/CTX/CAZ/CRO/FUR/COL/SIX	1	2.8

**Note:**

AMP, Ampicillin; CPM, Cefepime; CTX, Cefotaxime; CAZ, Ceftazidime, CRO, Ceftriaxone; FUR, Cefuroxime (sodium); COL, Colistin; S, Streptomycin, and SIX, Sulfisoxazole.

### Analysis of antimicrobial resistance genes (ARGs)

**Figure 1:**
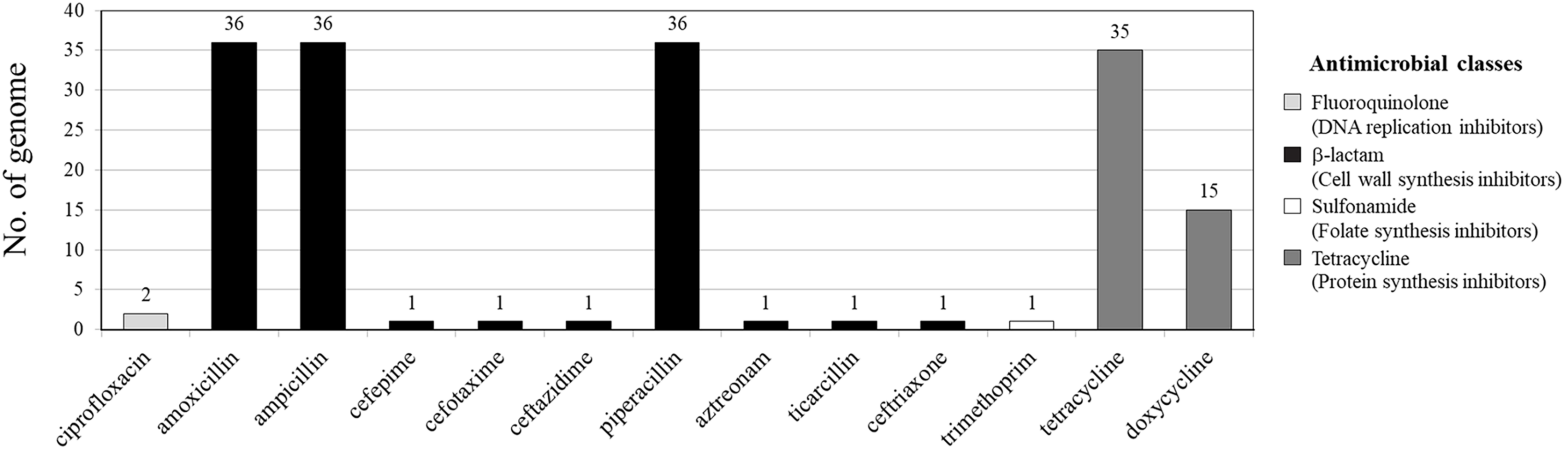
Antimicrobial resistance gene (ARG) profiles of 36 *Vibrio* *parahaemolyticus* genomes detected with ResFinder.

**Figure 2:**
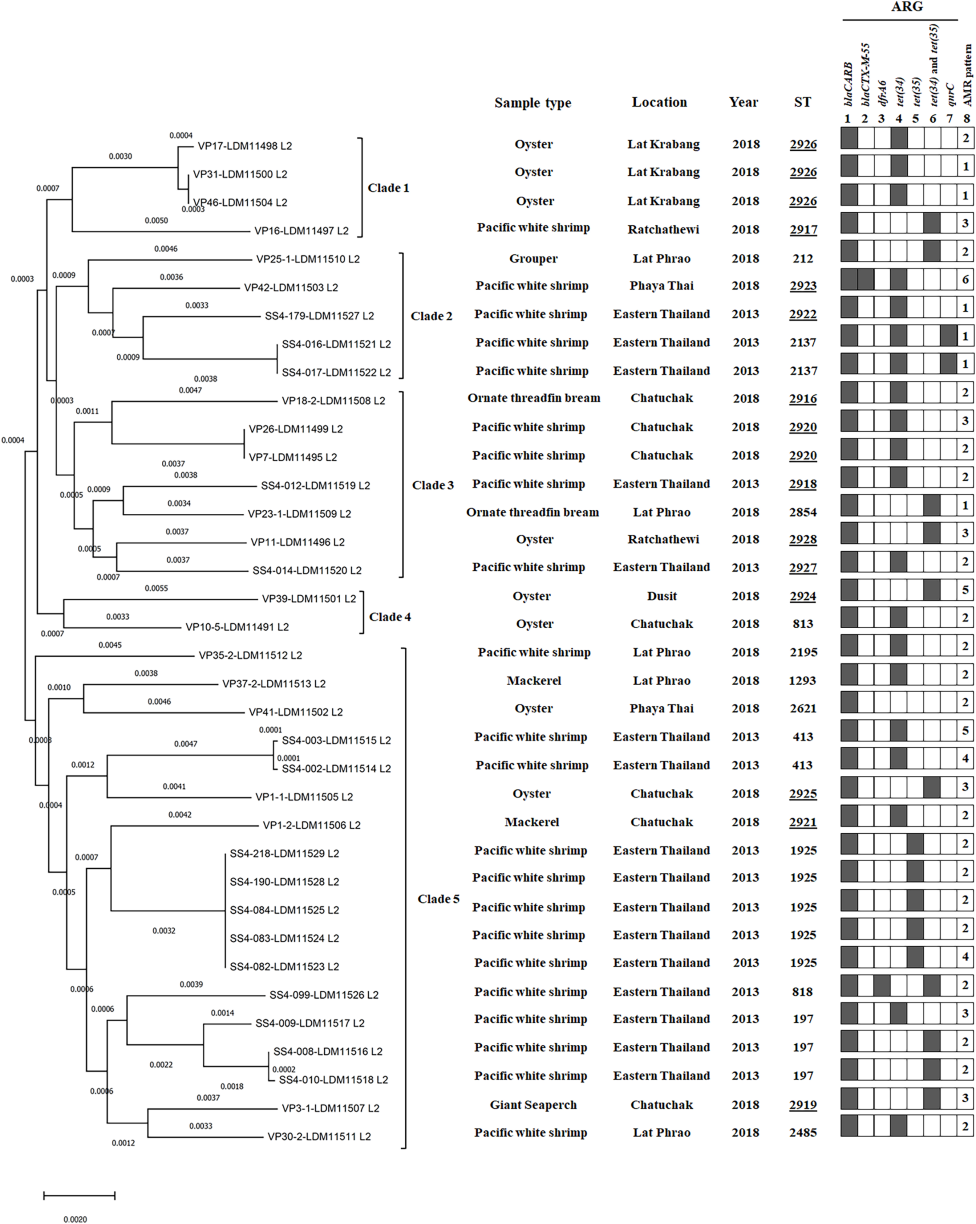
A phylogenomic tree from maximum likelihood analyzes of 81 bacterial core genes in *Vibrio* *parahaemolyticus* genomes and MLST types shows the relationship among 36 *V. parahaemolyticus* isolates. Antimicrobial resistance gene (ARG) profiles: (1) *blaCARB* gene causing resistance to amoxicillin, ampicillin and piperacillin; (2) *blaCTX-M-55* gene causing resistance to amoxicillin, ampicillin, piperacillin, cefepime, cefotaxime, ceftazidime, ceftriaxone, aztreonam, and ticarcillin; (3) *dfrA6* gene giving resistance to trimethoprim; (4, 5 and 6) *tet(34), tet(35)*, and *tet(34)* and *tet(35)* genes, respectively, causing resistance to tetracycline and doxycycline; (7) *qnrC* gene conferring ciprofloxacin resistance; (8) AMR patterns No. 1–6, as shown in Table 4. A filled box in column 1–7 specifies the identification of the particular gene. The sequence type (ST) with the underline represents the novel ST. The location column written eastern Thailand indicates the samples obtained from shrimp farms.

Among 36 *V. parahaemolyticus* genomes, 13 AMR genotypes and six ARGs were detected. Of six ARGs observed, *blaCARB* (100%, 36/36), *tet(34)* (83%, 30/36) and *tet(35)* (42%, 15/36) were the most common, followed by *qnrC* (6%, 2/36), *dfrA6* (3%, 1/36), and *blaCTX-M-55* (3%, 1/36) (Figs. 1 and 2). All 36 *V. parahaemolyticus* genomes carried at least one *blaCARB* gene (encoding for β-lactamase enzyme), causing resistance to amoxicillin, ampicillin, and piperacillin. Interestingly, VP42 carried *blaCTX-M-55* gene encoding for extended spectrum β-lactamase (ESBL) conferring resistance to amoxicillin, ampicillin, piperacillin, cefepime, cefotaxime, ceftazidime, ceftriaxone, aztreonam, and ticarcillin.

Of 36 *V. parahaemolyticus* isolates, 35 and 15 isolates harbored ARG of tetracycline and doxycycline, respectively. Of 35 isolates with tetracycline ARG, 20 isolates harbored *tet(34)*, 5 isolates harbored *tet(35)*, and 10 isolates harbored *tet(34)* and *tet(35)*. All 15 isolates with doxycycline ARG harbored *tet(35)*. SS4-099 isolated from Pacific white shrimp carried *dfrA6* gene giving resistance to trimethoprim. SS4-016-and SS4-017 isolated from Pacific white shrimp carried *qnrC* gene giving resistance to ciprofloxacin (Fig. 2).

### Multilocus sequence type (MLST) analysis

MLST analysis revealed 25 different sequence types (STs) of 36 *V. parahaemolyticus* isolates (Table S6). Twelve STs (48%, 12/25) of 20 isolates were previously reported in pubMLST Database, whereas 13 novel STs (ST2916-ST2928, 52%, 13/25) of 16 isolates obtained in this study were submitted to PubMLST/*V. parahaemolyticus* database (http://pubmlst.org/vparahaemolyticus). GenBank accession numbers of 36 *V. parahaemolyticus* genomes are shown in Table S2. Among the novel STs (16 isolates), ST2926 was predominant (three isolates), followed by ST2920 (two isolates). Most of the novel STs were identified from marketed seafood (10 STs) and the rest three novel STs (ST2918, ST2922, and ST2927) were identified from shrimp farms. The most common STs detected in this study were ST1925 (20%, 5/25), ST197 (12%, 3/25), ST2926 (12%, 3/25), ST413 (8%, 2/25), ST2137 (8%, 2/25), and ST2920 (8%, 2/25) (Table S6).

### Phylogenomic analysis

A phylogenomic tree from maximum likelihood analyzes of 81 bacterial core genes in 36 *V. parahaemolyticus* genomes exhibited five distinct clades (Fig. 2). All four isolates in clade 1 were identified as novel STs, ST2926 (VP17, VP31, and VP46) and ST2917 (VP16) and the other 11 novel STs were distributed to clade 2–5 (12 isolates). ST2926, ST2137, and ST2920 were identified in three isolates of clade 1, 2 isolates of clade 2 and 2 isolates of clade 3, respectively. ST197, ST413, ST1925, and ST818 were identified in 3, 2, 5, and 1 isolates of clade 5, respectively. These results exhibited a close relationship among *V. parahaemolyticus* strains which were identified from the same seafood types collected in the same year and from the same location. A close association among the strains isolated from the same seafood types collected in the same year but from different locations was also found in clade 4 (VP39 and VP10/5). Additionally, a close relationship among the strains isolated from different types of seafood in different years and different locations was exhibited in clade 3 (SS4-012 and VP23/1 as well as VP11 and SS4-014 isolates). SS4-016 and SS4-017 with the same ST2137 carrying *qnrC* isolated from farmed shrimp in eastern Thailand and VP42 (ST2923), carrying *blaCTX-M-55* isolated from shrimp sold in Bangkok were in the same clade 2. These results indicated the association between these isolates from different geographical regions. Moreover, SS4-099 isolate carrying *dfrA6* was in clade 5 and is distinct from SS4-016 and SS4-017 isolates carrying *qnrC* in clade 2. These three isolates were isolated from farmed shrimp in eastern Thailand indicating a distance relationship among these strains isolated from the same region.

## Discussion

In this study, all *V. parahaemolyticus* strains isolated from seafood purchased in Bangkok and collected in eastern Thailand belonged to the old O3:K6 strains or non-pandemic strains. In contrast, 12 strains isolated from 302 seafood samples purchased in southern Thailand were pandemic strains (Vuddhakul et al., 2006). Jingjit et al. (2021) also identified 15 pandemic *V. parahaemolyticus* strains from 51 seafood-isolated *V. parahaemolyticus* strains obtained from southern Thailand (Jingjit et al., 2021). These pandemic strains were detected from hard clams, mussels, and cockles (Vuddhakul et al., 2006; Jingjit et al., 2021) Mussels, and cockles were also purchased from markets in this study. The discrepancies in pandemic strain occurrences are likely due to the differences in geographical regions for seafood sample collection. All seafood samples in this study were purchased from fresh markets and supermarkets in Bangkok and collected from farms in the upper Gulf of Thailand (central and eastern Thailand), while seafood samples in previous studies were collected from the lower Gulf of Thailand (southern Thailand). The O3:K6 pandemic strain had been firstly identified in 1998 at Hat Yai Hospital in southern Thailand (Matsumoto et al., 2000), later pandemic *V. parahaemolyticus* strains were reported from seafood samples in the same area (Vuddhakul et al., 2006; Jingjit et al., 2021) indicating that pandemic strains have established ecological niches in this region.

The incidence of *V. parahaemolyticus* in this study is somewhat low at 20%, compared to previous reports which seafood samples were also purchased in Bangkok, Thailand at 58% (Thitipetchrakul, Somyoonsup & Hatayananont, 2016) and at 59% (Atwill & Jeamsripong, 2021). This is likely due to the differences in seafood types, market types and sample storage condition between this study and previous studies. *V. parahaemolyticus* contamination was found more often in seafood purchased from fresh markets than those from supermarkets (Zhang et al., 2016; Martinez-Urtaza & Baker-Austin, 2020) and in refrigerated products more than frozen products (Caburlotto et al., 2016). In this study, more different seafood types were purchased from fresh markets and supermarkets of which some samples were frozen seafood whereas the study by Atwill & Jeamsripong (2021) used chilled seafood samples consisting of Pacific white shrimp, oysters, blood cockles, and Asian seabass purchased form open-air retail fresh markets (Atwill & Jeamsripong, 2021). Boiled crab meats from fresh and fair markets were used for *V. parahaemolyticus* surveillance (Thitipetchrakul, Somyoonsup & Hatayananont, 2016). Thus, the incidence of *V. parahaemolyticus* in this study was likely lower than those of previous reports.

Our results showed that none of 50 *V. parahaemolyticus* isolates harbored T3SS2 genes while VPaI-7 gene (VP1321) was present in two isolates. The absence of T3SS2 genes in two isolates positive for VPaI-7 is probably due to the partial loss of T3SS2 genes as previously reported (Chao et al., 2009). VPaI-7 on chromosome two typically encodes for two *tdh* (*tdh1* and *tdh2*) genes and a set of T3SS2 genes which are virulence genes (Makino et al., 2003; Matsuda et al., 2020). *V. parahaemolyticus* harboring T3SS2 genes has been linked to clinical cases of inflammatory gastroenteritis (Makino et al., 2003; Hiyoshi et al., 2011). Although most previous studies reported the presence of T3SS2 only in highly virulent strains; however, T3SS2 has also been observed in a non-virulent strain (Meador et al., 2007). This study focused on VP1321 ORF of VPaI-7 and two T3SS2 genes (VP1346 and VP1367) as they were observed in all the pandemic strains reported previously (Chao et al., 2009). Although, none of tested seafood samples purchased in Bangkok and collected in eastern Thailand were pandemic strains, detection of VPaI-7 in non-pandemic-pathogenic *V. parahaemolyticus* strains provided the importance of seafood safety through consumer health awareness and practices. Strong biofilm formation ability has been linked to environmental survival ability, infectivity, and transmissibility of antibiotic-resistance microorganisms to humans (Elexson et al., 2014; Mizan, Jahid & Ha, 2015). Our results indicated that all 50 *V. parahaemolyticus* isolates consisting of 21 pathogenic and 29 non-pathogenic strains harbored three biofilm-associated genes (VP0950, VP0952, and VP0962). Under proper circumstances, these genes could be activated and expressed leading to biofilm formation in *V. parahaemolyticus* contaminated seafood that could cause a great threat to human health and economic values.

Our study demonstrated a high prevalence of AMR phenotypes among the 36 *V. parahaemolyticus* isolates for ampicillin (83%, 30/36) and colistin (100%, 36/36), which was in consistent with the frequent outbreaks of ampicillin- and colistin-resistant *V. parahaemolyticus* isolates reported in the past 5 years (Lopatek, Wieczorek & Osek, 2018; Dahanayake et al., 2020; Mok et al., 2021; Nishino et al., 2021; Vu et al., 2022). The *blaCARB* gene was present in all 36 *V. parahaemolyticus* isolates, confirming ampicillin-resistant phenotypes and susceptible phenotypes to amoxicillin/clavulanic acid, ampicillin/sulbactam, and piperacillin/tazobactam. The high distribution of *blaCARB* gene is similar to a previous study which found that all 30 *V. parahaemolyticus* isolates obtained from shrimp samples harbored *blaCARB* (Hossain et al., 2020). Consistently with our findings, a report by Chiou, Li & Chen (2015) suggested an intrinsic resistance of ampicillin in *V. parahaemolyticus*. The observed amoxicillin resistance phenotypes in all isolates and the higher prevalence of *tet(34)*, conferring resistance to oxytetracycline (Nonaka & Suzuki, 2002), than that of *tet(35)* are in agreement with the excessive uses of amoxicillin and oxytetracycline in aquaculture. These two antimicrobial agents have been permitted for use in Asian aquaculture industries including Thailand (Yano et al., 2014; AAHRDD, 2022). Based on this finding, we recommend that antimicrobial use in aquaculture should follow guidelines recommended by Food and Drug Administration (FDA) of Thailand including using antimicrobials based on clinical diagnosis and using narrow-spectrum antimicrobials. Rotation of permitted antimicrobials should also be done to decrease the antimicrobial pressure due to the dominant use. The *blaCTX-M-55* gene was identified in VP42 isolate confirming phenotypic resistance against cefuroxime, cefotaxime, ceftazidime, ceftriaxone, and cefepime. The low distribution of *blaCTX-M-55* is in agreement with a study conducted in China which *blaCTX-M-55* was found in 2% (2/116) of *V. parahaemolyticus* strains isolated from shrimp samples (Zheng et al., 2019). Detection of cephalosporins resistant isolates causes concern as cephalosporins are among the β-lactams currently used as the last line of antibiotics to treat *V. parahaemolyticus* infections (Ashrafudoulla et al., 2021; Dutta et al., 2021). Our results showed significant correlations between phenotypes and genotypes that conferred resistance to ampicillin (*blaCARB*) and cephalosporins (*blaCTX-M-55*). No genotypic resistance was observed for colistin, sulfisoxazole, and streptomycin. It is possible that ARGs are mobile genetic elements which may not be detected by a short-read sequencing method (Christaki, Marcou & Tofarides, 2020; Dutta et al., 2021). No phenotypic resistance was observed despite genotypic resistance for trimethoprim, ciprofloxacin, doxycycline, and tetracycline. It is plausible that these genes were silenced or expressed at low resistance level below the interpretive breakpoint utilized. Under suitable circumstances, these genes could be highly expressed and/or transferred horizontally among bacteria posing the risk of AMR and/or MDR phenotypes (Dutta et al., 2021). The discrepancy between phenotypic and genotypic resistance observed here was similar to the previous finding of pathogenic *V. parahaemolyticus* in seafood samples (Lou et al., 2016). Antibiotic misuse, warm temperature, acid-base and organic contamination can directly activate the expression of ARGs and virulence genes and also affect horizontal gene transfer (Guijarro et al., 2015; Deng et al., 2020). Thus, the occurrence of tetracycline, doxycycline and ciprofloxacin resistance genes is a major concern since they are the antibiotics of choice for treatment of severe or prolonged illnesses of *V. parahaemolyticus* infection (Tan et al., 2020; Ashrafudoulla et al., 2021; Dutta et al., 2021). The high resistance for ampicillin and colistin observed here suggests an alarming trend of widespread ampicillin and colistin resistance which compromise treatment efficacy for *V. parahaemolyticus* infection. It is important to note that due to limited number of isolates, specific locations, and samples per seafood species, years and market types in this study, it might be very challenging to conclude the current AMR situation of *V. parahaemolyticus* in Thailand. However, the high resistance to ampicillin and high susceptibility to cephalosporins found in seafood *V. parahaemolyticus* confirms previous findings from studies conducted in Thailand and other Asian countries (Elmahdi, DaSilva & Parveen, 2016; Palamae et al., 2022).

MLST analysis demonstrated a high number (25) of STs and particularly 13 novel STs among the 36 isolates suggesting a high genetic variation of Thai *V. parahaemolyticus* isolates. The relationship among the strains with specific ST found in the same seafood type collected from the same location in the same year revealed a close relationship among these strains suggesting cross-contamination during harvesting or handling seafood at the same processing site due to poor hygiene practices. Thus, local interventions should be addressed including using disinfected seawater or potable water to wash and process. seafood, wearing gloves during processing and keeping seafood at ≤10 °C during distribution and storage (Hara-Kudo & Kumagai, 2014). A close association among the *V. parahaemolyticus* strains isolated from the same type of seafood from different locations in the same year or different types of seafood from distinct locations and sampling years could indicated either the possibility of cross-contamination during food processing steps of supply chains or persistence of these strains in the environment. Overall, MLST analysis revealed that *V. parahaemolyticus* strains in Thailand were geographically distinct and genetically diverse. The strains isolated from marketed samples were more diverse than those from shrimp farms. These observations suggest the potential cross-contamination of *V. parahaemolyticus* during seafood processing steps and distribution chains including a contaminated container for transporting and improper handling. In contrast, *V. parahaemolyticus* in farmed shrimp is mainly derived from environmental contamination including estuarine water and water sediment at the cultivation site (Lovell, 2017). Several decontamination techniques are available to effectively reduce the number of *V. parahaemolyticus* without compromising the flavor, texture, and nutritional of seafood products. The decontamination techniques comprise chemical techniques (antibiotics, disinfectants and natural organic treatments), physical techniques (high-pressure processing, ozonation, irradiation, refrigeration and seafood suspension), and biological techniques (bacteriophage and probiotic treatments) (Ramos et al., 2012; Hara-Kudo et al., 2013; Wang et al., 2013; Ronholm, Lau & Banerjee, 2016; Zhang et al., 2018; Kontominas et al., 2021).

## Conclusions

Our findings reveal that all *V. parahaemolyticus* isolates obtained from seafood in Bangkok and eastern Thailand are non-pandemic strains; some of them contain pathogenic potential genes. The presence of the first-line antimicrobial resistance genes of tetracyclines, doxycycline and ciprofloxacin raises a major concern of inducible gene expression and/or horizontal gene transfer among bacteria. The widespread of MDR *V. parahaemolyticus* confirms the emergence of AMR problems in seafood. Thus, monitoring of AMR of *V. parahaemolyticus* in seafood is highly recommended to tackle AMR problem and provide useful information for therapeutic treatment in humans. Based on our findings, antimicrobial use to treat *V. parahaemolyticus* infection in aquaculture should restrictedly follow guidelines recommended by FDA of Thailand to avoid AMR and MDR problems. Moreover, most of *V. parahaemolyticus* strains possess new STs suggesting high genetic diversity of the isolates. *V. parahaemolyticus* diversity in marketed samples suggests the cross-contamination possibility among samples in seafood production chains. Overall, our findings highlight the importance of a surveillance program to help strengthen safety guidelines of seafood production and promote public health awareness among health professional and consumers.

## Supplemental Information

10.7717/peerj.15283/supp-1 Supplemental Information 1 Antimicrobial categories, agents and concentrations used in this study.

10.7717/peerj.15283/supp-2 Supplemental Information 2 GenBank accession numbers of 36 *Vibrio parahaemolyticus* genomes. The genome sequences of 36 *Vibrio parahaemolyticus* isolates in this study are available at NCBI database: PRJNA859558 (https://www.ncbi.nlm.nih.gov/bioproject/PRJNA859558). Each assembled genome sequence is assigned to BioSample.

10.7717/peerj.15283/supp-3 Supplemental Information 3 Distribution of pandemic and non-pandemic strains, and potential pathogenic genes of 50 *Vibrio* *parahaemolyticus* isolates.

10.7717/peerj.15283/supp-4 Supplemental Information 4 Antimicrobial categories, agents, and susceptibility of 36 *Vibrio* *parahaemolyticus* isolated from seafood in Thailand.

10.7717/peerj.15283/supp-5 Supplemental Information 5 Antimicrobial resistance (AMR) patterns and multiple antibiotic resistance (MAR) indexes of 36 *Vibrio parahaemolyticus*isolates.

10.7717/peerj.15283/supp-6 Supplemental Information 6 Distribution of 25 sequence types (STs) with 12 known and 13 novel STs of 36 *Vibrio* *parahaemolyticus* isolated from seafood in Thailand.

10.7717/peerj.15283/supp-7 Supplemental Information 7 The raw data for minimum inhibitory concentration (MIC) values of the 36 *Vibrio parahaemolyticus* isolates. The raw data of the antimicrobial susceptibility test performed on the 36 *Vibrio parahaemolyticus* isolates against 27 agents from 5 antimicrobial categories.

## Additional Information and Declarations

### DNA Deposition

The following information was supplied regarding the deposition of DNA sequences: The genome sequences of 36 *Vibrio parahaemolyticus* isolates in this study are available at the NCBI database: PRJNA859558. Each assembled genome sequence is assigned to BioSample. The genome sequences are available at GenBank: JANFKC000000000 to JANFKZ000000000 and JANFLA000000000 to JANFLO000000000 as shown in Table S2.

### Data Availability

The following information was supplied regarding data availability: The raw data for minimum inhibitory concentration (MIC) values of 36 *Vibrio parahaemolyticus* isolates against 27 agents from 5 antimicrobial categories are available in the Supplemental Files.
