# Supplementary material for: Incidence, genetic diversity, and antimicrobial resistance profiles of Vibrio parahaemolyticus in seafood in Bangkok and eastern Thailand
Source: PeerJ. 2023 May 11;11:e15283. doi: 10.7717/peerj.15283 (PMC10183165; doi:10.7717/peerj.15283)
Supplement: Supplemental Information 1 [file peerj-11-15283-s001.docx]

**Table S1** Antimicrobial categories, agents and concentrations used in this study

^#^ THAN1F and CMV4AGNF are the dehydrated 96-well Gram negative MIC microtiter plates.

| **Antimicrobial category** | **Antimicrobial agent** | **Concentration (μg)** | |
| --- | --- | --- | --- |
|  |  | ^#^**THAN1F** | ^#^**CMV4AGNF** |
| **DNA replication inhibitor**  Fluoroquinolones | Ciprofloxacin  Levofloxacin  Nalidixic Acid | 0.06-2  0.06-8  - | 0.015-4  -  0.5-32 |
| **Cell wall synthesis inhibitor**  β-lactams  Penicillins/β-lactamase inhibitors  Carbapenems  Cephalosporins | Ampicillin  Amoxicillin/Clavulanic Acid  Ampicillin/Sulbactam  Piperacillin/Tazobactam  Doripenem  Ertapenem  Imipenem  Meropenem  Cefoxitin  Cefuroxime (sodium)  Cefotaxime  Ceftazidime  Ceftriaxone  Cefepime | 8-32  4/2-16/8  4/2-16/8  8/4-64/4  0.5-4  0.5-4  0.5-8  0.5-8  4-16  8-16  1-32  1-32  0.5-32  1-32 | 1-32  1/0.5-32/16  -  -  -  -  -  0.06-4  0.5-32  -  -  -  0.5-64  - |
| **Outer cell membrane disruptor**  Polymyxins | Colistin | 1-4 | - |
| **Folate synthesis inhibitor**  Sulfonamides | Sulfisoxazole  Trimethoprim/Sulfamethoxazole | -  1/19-4/76 | 16-256  0.12/2.38-4/76 |
| **Protein synthesis inhibitor**  **30S subunit**  Aminoglycosides  Tetracyclines  **50S subunit**  Phenicols  Macrolides | Amikacin  Gentamicin  Netilmicin  Streptomycin  Tetracyclines  Chloramphenicol  Azithromycin | 8-32  2-8  8-16  -  -  -  - | -  0.25-16  -  2-64  4-32  2-32  0.25-32 |
