# Supplementary material for: Incidence, genetic diversity, and antimicrobial resistance profiles of Vibrio parahaemolyticus in seafood in Bangkok and eastern Thailand
Source: PeerJ. 2023 May 11;11:e15283. doi: 10.7717/peerj.15283 (PMC10183165; doi:10.7717/peerj.15283)
Supplement: Supplemental Information 2 — The genome sequences of 36 Vibrio parahaemolyticus isolates in this study are available at NCBI database: PRJNA859558 (https://www.ncbi.nlm.nih.gov/bioproject/PRJNA859558). Each assembled genome sequence is assigned to BioSample. [file peerj-11-15283-s002.docx]

**Table S2** GenBank accession numbers of 36 *Vibrio parahaemolyticus* genomes

The genome sequences of 36 *Vibrio* *parahaemolyticus* isolates in this study are submitted to NCBI database under the BioProject **PRJNA859558** (<https://www.ncbi.nlm.nih.gov/bioproject/PRJNA859558>). Each assembled genome sequence is assigned to BioSample.

| **Genome sample** | **NCBI BioSample accession number** | **NCBI Genome accession number** |
| --- | --- | --- |
| VP7 | SAMN29790235 (https://www.ncbi.nlm.nih.gov/biosample/SAMN29790235) | JANFKC000000000 (https://www.ncbi.nlm.nih.gov/nuccore/JANFKC000000000) |
| VP46 | SAMN29790236 (https://www.ncbi.nlm.nih.gov/biosample/SAMN29790236) | JANFKD000000000 (https://www.ncbi.nlm.nih.gov/nuccore/JANFKD000000000) |
| VP42 | SAMN29790237 (https://www.ncbi.nlm.nih.gov/biosample/SAMN29790237) | JANFKE000000000 (https://www.ncbi.nlm.nih.gov/nuccore/JANFKE000000000) |
| VP41 | SAMN29790238 (https://www.ncbi.nlm.nih.gov/biosample/SAMN29790238) | JANFKF000000000 (https://www.ncbi.nlm.nih.gov/nuccore/JANFKF000000000) |
| VP39 | SAMN29790239 (https://www.ncbi.nlm.nih.gov/biosample/SAMN29790239) | JANFKG000000000 (https://www.ncbi.nlm.nih.gov/nuccore/JANFKG000000000) |
| VP37-2 | SAMN29790240 (https://www.ncbi.nlm.nih.gov/biosample/SAMN29790240) | JANFKH000000000 (https://www.ncbi.nlm.nih.gov/nuccore/JANFKH000000000) |
| VP35-2 | SAMN29790241 (https://www.ncbi.nlm.nih.gov/biosample/SAMN29790241) | JANFKI000000000 (https://www.ncbi.nlm.nih.gov/nuccore/JANFKI000000000) |
| VP31 | SAMN29790242 (https://www.ncbi.nlm.nih.gov/biosample/SAMN29790242) | JANFKJ000000000 (https://www.ncbi.nlm.nih.gov/nuccore/JANFKJ000000000) |
| VP3-1 | SAMN29790243 (https://www.ncbi.nlm.nih.gov/biosample/SAMN29790243) | JANFKK000000000 (https://www.ncbi.nlm.nih.gov/nuccore/JANFKK000000000) |
| VP30-2 | SAMN29790244 (https://www.ncbi.nlm.nih.gov/biosample/SAMN29790244) | JANFKL000000000 (https://www.ncbi.nlm.nih.gov/nuccore/JANFKL000000000) |
| VP26 | SAMN29790245 (https://www.ncbi.nlm.nih.gov/biosample/SAMN29790245) | JANFKM000000000 (https://www.ncbi.nlm.nih.gov/nuccore/JANFKM000000000) |
| VP25-1 | SAMN29790246 (https://www.ncbi.nlm.nih.gov/biosample/SAMN29790246) | JANFKN000000000 (https://www.ncbi.nlm.nih.gov/nuccore/JANFKN000000000) |
| VP23-1 | SAMN29790247 (https://www.ncbi.nlm.nih.gov/biosample/SAMN29790247) | JANFKO000000000 (https://www.ncbi.nlm.nih.gov/nuccore/JANFKO000000000) |
| VP18-2 | SAMN29790251 (https://www.ncbi.nlm.nih.gov/biosample/SAMN29790251) | JANFKS000000000 (https://www.ncbi.nlm.nih.gov/nuccore/JANFKS000000000) |
| VP17 | SAMN29790252 (https://www.ncbi.nlm.nih.gov/biosample/SAMN29790252) | JANFKT000000000 (https://www.ncbi.nlm.nih.gov/nuccore/JANFKT000000000) |
| VP16 | SAMN29790253 (https://www.ncbi.nlm.nih.gov/biosample/SAMN29790253) | JANFKU000000000 (https://www.ncbi.nlm.nih.gov/nuccore/JANFKU000000000) |
| VP1-2 | SAMN29790254 (https://www.ncbi.nlm.nih.gov/biosample/SAMN29790254) | JANFKV000000000 (https://www.ncbi.nlm.nih.gov/nuccore/JANFKV000000000) |
| VP1-1 | SAMN29790255 (https://www.ncbi.nlm.nih.gov/biosample/SAMN29790255) | JANFKW000000000 (https://www.ncbi.nlm.nih.gov/nuccore/JANFKW000000000) |
| VP11 | SAMN29790256 (https://www.ncbi.nlm.nih.gov/biosample/SAMN29790256) | JANFKX000000000 (https://www.ncbi.nlm.nih.gov/nuccore/JANFKX000000000) |
| VP10-5 | SAMN29790257 (https://www.ncbi.nlm.nih.gov/biosample/SAMN29790257) | JANFKY000000000 (https://www.ncbi.nlm.nih.gov/nuccore/JANFKY000000000) |
| SS4-218 | SAMN29790258 (https://www.ncbi.nlm.nih.gov/biosample/SAMN29790258) | JANFKZ000000000 (https://www.ncbi.nlm.nih.gov/nuccore/JANFKZ000000000) |
| SS4-190 | SAMN29790259 (https://www.ncbi.nlm.nih.gov/biosample/SAMN29790259) | JANFLA000000000 (https://www.ncbi.nlm.nih.gov/nuccore/JANFLA000000000) |
| SS4-179 | SAMN29790260 (https://www.ncbi.nlm.nih.gov/biosample/SAMN29790260) | JANFLB000000000 (https://www.ncbi.nlm.nih.gov/nuccore/JANFLB000000000) |
| SS4-099 | SAMN29790261 (https://www.ncbi.nlm.nih.gov/biosample/SAMN29790261) | JANFLC000000000 (https://www.ncbi.nlm.nih.gov/nuccore/JANFLC000000000) |
| SS4-084 | SAMN29790262 (https://www.ncbi.nlm.nih.gov/biosample/SAMN29790262) | JANFLD000000000 (https://www.ncbi.nlm.nih.gov/nuccore/JANFLD000000000) |
| SS4-083 | SAMN29790263 (https://www.ncbi.nlm.nih.gov/biosample/SAMN29790263) | JANFLE000000000 (https://www.ncbi.nlm.nih.gov/nuccore/JANFLE000000000) |
| SS4-082 | SAMN29790264 (https://www.ncbi.nlm.nih.gov/biosample/SAMN29790264) | JANFLF000000000 (https://www.ncbi.nlm.nih.gov/nuccore/JANFLF000000000) |
| SS4-017 | SAMN29790265 (https://www.ncbi.nlm.nih.gov/biosample/SAMN29790265) | JANFLG000000000 (https://www.ncbi.nlm.nih.gov/nuccore/JANFLG000000000) |
| SS4-016 | SAMN29790266 (https://www.ncbi.nlm.nih.gov/biosample/SAMN29790266) | JANFLH000000000 (https://www.ncbi.nlm.nih.gov/nuccore/JANFLH000000000) |
| SS4-014 | SAMN29790267 (https://www.ncbi.nlm.nih.gov/biosample/SAMN29790267) | JANFLI000000000 (https://www.ncbi.nlm.nih.gov/nuccore/JANFLI000000000) |
| SS4-012 | SAMN29790268 (https://www.ncbi.nlm.nih.gov/biosample/SAMN29790268) | JANFLJ000000000 (https://www.ncbi.nlm.nih.gov/nuccore/JANFLJ000000000) |
| SS4-010 | SAMN29790269 (https://www.ncbi.nlm.nih.gov/biosample/SAMN29790269) | JANFLK000000000 (https://www.ncbi.nlm.nih.gov/nuccore/JANFLK000000000) |
| SS4-009 | SAMN29790270 (https://www.ncbi.nlm.nih.gov/biosample/SAMN29790270) | JANFLL000000000 (https://www.ncbi.nlm.nih.gov/nuccore/JANFLL000000000) |
| SS4-008 | SAMN29790271 (https://www.ncbi.nlm.nih.gov/biosample/SAMN29790271) | JANFLM000000000 (https://www.ncbi.nlm.nih.gov/nuccore/JANFLM000000000) |
| SS4-003 | SAMN29790272 (https://www.ncbi.nlm.nih.gov/biosample/SAMN29790272) | JANFLN000000000 (https://www.ncbi.nlm.nih.gov/nuccore/JANFLN000000000) |
| SS4-002 | SAMN29790273 (https://www.ncbi.nlm.nih.gov/biosample/SAMN29790273) | JANFLO000000000 (https://www.ncbi.nlm.nih.gov/nuccore/JANFLO000000000) |

Please note that the submission is set to release 31^st^ April 2023 date or upon publication, whichever is first.
