# Supplementary material for: Incidence, genetic diversity, and antimicrobial resistance profiles of Vibrio parahaemolyticus in seafood in Bangkok and eastern Thailand
Source: PeerJ. 2023 May 11;11:e15283. doi: 10.7717/peerj.15283 (PMC10183165; doi:10.7717/peerj.15283)
Supplement: Supplemental Information 3 [file peerj-11-15283-s003.docx]

**Table S3**  Distribution of pandemic and non-pandemic strains, and potential pathogenic genes of 50 *Vibrio parahaemolyticus* isolates

| **Isolate code** | ***toxR*** | **^1^Non-pandemic** | **^2^Pandemic** | **VPaI-7**  **VP1321** | **VpaI-7 (T3SS2)** | | **Biofilm** | | |
| --- | --- | --- | --- | --- | --- | --- | --- | --- | --- |
|  |  |  |  |  | **VP1346** | **VP1367** | **VP0950** | **VP0952** | **VP0962** |
| **VP 10/5** | + | + | − | − | − | − | + | + | + |
| VP 7 | + | + | − | − | − | − | + | + | + |
| VP 11 | + | + | − | − | − | − | + | + | + |
| VP 16 | + | + | − | − | − | − | + | + | + |
| VP 17 | + | + | − | − | − | − | + | + | + |
| VP 26 | + | + | − | − | − | − | + | + | + |
| VP 31 | + | + | − | − | − | − | + | + | + |
| VP 39 | + | + | − | − | − | − | + | + | + |
| VP 41 | + | + | − | − | − | − | + | + | + |
| VP 42 | + | + | − | − | − | − | + | + | + |
| VP 46 | + | + | − | − | − | − | + | + | + |
| VP1/1 | + | + | − | − | − | − | + | + | + |
| VP 1/2 | + | + | − | − | − | − | + | + | + |
| VP 3/1 | + | + | − | − | − | − | + | + | + |
| VP 18/2 | + | + | − | − | − | − | + | + | + |
| VP 23/1 | + | + | − | − | − | − | + | + | + |
| VP 25/1 | + | + | − | − | − | − | + | + | + |
| VP 30/2 | + | + | − | − | − | − | + | + | + |
| VP 35/2 | + | + | − | − | − | − | + | + | + |
| VP 37/2 | + | + | − | − | − | − | + | + | + |
| SS4-002 | + | + | − | − | − | − | + | + | + |
| **SS4-003** | + | + | − | − | − | − | + | + | + |
| **SS4-008** | + | + | − | − | − | − | + | + | + |
| SS4-009 | + | + | − | − | − | − | + | + | + |
| SS4-010 | + | + | − | − | − | − | + | + | + |
| **SS4-012** | + | + | − | − | − | − | + | + | + |
| SS4-014 | + | + | − | − | − | − | + | + | + |
| **SS4-016** | + | + | − | − | − | − | + | + | + |
| **SS4-017** | + | + | − | − | − | − | + | + | + |
| **SS4-082** | + | + | − | − | − | − | + | + | + |
| **SS4-083** | + | + | − | − | − | − | + | + | + |
| **SS4-084** | + | + | − | − | − | − | + | + | + |
| **SS4-099** | + | + | − | − | − | − | + | + | + |
| **SS4-179** | + | + | − | − | − | − | + | + | + |
| **SS4-190** | + | + | − | − | − | − | + | + | + |
| **SS4-218** | + | + | − | − | − | − | + | + | + |
| F1SS01 | + | + | − | − | − | − | + | + | + |
| F1BS02 | + | + | − | − | − | − | + | + | + |
| **F1MS03** | + | + | − | − | − | − | + | + | + |
| **F2BSC01** | + | + | − | − | − | − | + | + | + |
| **F2CK02** | + | + | − | + | − | − | + | + | + |
| **F2OY03** | + | + | − | − | − | − | + | + | + |
| **F3CK01** | + | + | − | + | − | − | + | + | + |
| S1WS01 | + | + | − | − | − | − | + | + | + |
| S2WS01 | + | + | − | − | − | − | + | + | + |
| **S3WSB01** | + | + | − | − | − | − | + | + | + |
| S3WSB02 | + | + | − | − | − | − | + | + | + |
| **S3BP03** | + | + | − | − | − | − | + | + | + |
| **S3OY09** | + | + | − | − | − | − | + | + | + |
| S6SS01 | + | + | − | − | − | − | + | + | + |

+, presence of gene sequence; −, absence of gene sequence; ^1^Determined by the presence of *toxRS/*old genes; ^2^Determined by the presence of both *tdh* and *toxRS/*new; Bold letters indicate pathogenic strains. The underline indicates the samples excluded from an antimicrobial susceptibility test.
