# Supplementary material for: Incidence, genetic diversity, and antimicrobial resistance profiles of Vibrio parahaemolyticus in seafood in Bangkok and eastern Thailand
Source: PeerJ. 2023 May 11;11:e15283. doi: 10.7717/peerj.15283 (PMC10183165; doi:10.7717/peerj.15283)
Supplement: Supplemental Information 4 [file peerj-11-15283-s004.docx]

**Table S4** Antimicrobial categories, agents, and susceptibility of 36 *Vibrio parahaemolyticus* isolated from seafood in Thailand

| **Antimicrobial category** | **Antimicrobial agent** | **Number of isolates (%)** | | |
| --- | --- | --- | --- | --- |
|  |  | **Sensitive** | **Intermediate** | **Resistant** |
| **DNA replication inhibitor**  Fluoroquinolones | Ciprofloxacin  Levofloxacin  Nalidixic Acid | 36/36 (100)  36/36 (100)  36/36 (100) | -  -  - | -  -  - |
| **Cell wall synthesis inhibitor**  β-lactams  Penicillins/β-lactamase inhibitors  Carbapenems  Cephalosporins | Ampicillin  Amoxicillin/Clavulanic Acid  Ampicillin/Sulbactam  Piperacillin/Tazobactam  Doripenem  Ertapenem  Imipenem  Meropenem  Cefoxitin 2^nd^ gen.  Cefuroxime (sodium) 2^nd^ gen.  Cefotaxime 3^rd^ gen.  Ceftazidime 3^rd^ gen.  Ceftriaxone 3^rd^ gen.  Cefepime 4^th^ gen. | -  36/36 (100)  36/36 (100)  36/36 (100)  36/36 (100)  36/36 (100)  36/36 (100)  36/36 (100)  35/36 (97)  4/36 (11)  35/36 (97)  35/36 (97)  35/36 (97)  35/36 (97) | 6/36 (17)  -  -  -  -  -  -  -  1/36 (3)  29/36 (81)  -  -  -  - | 30/36 (83)  -  -  -  -  -  -  -  -  3/36 (8)  1/36 (3)  1/36 (3)  1/36 (3)  1/36 (3) |
| **Outer cell membrane disruptors**  Polymyxins | Colistin | - | - | 36/36 (100) |
| **Folate synthesis inhibitors**  Sulfonamides | Sulfisoxazole  Trimethoprim/Sulfamethoxazole | 33/36 (92)  36/36 (100) | -  - | 3/36 (8)  - |
| **Protein synthesis inhibitors**  **30S subunit**  Aminoglycosides  Tetracyclines  **50S subunit**  Phenicols  Macrolides | Amikacin  Gentamicin  Netilmicin  Streptomycin  Tetracyclines  Chloramphenicol  Azithromycin | 36/36 (100)  36/36 (100)  36/36 (100)  30/36 (83)  36/36 (100)  36/36 (100)  36/36 (100) | -  -  -  -  -  - | -  -  -  6/36 (17)  -  -  - |
