# Supplementary material for: Incidence, genetic diversity, and antimicrobial resistance profiles of Vibrio parahaemolyticus in seafood in Bangkok and eastern Thailand
Source: PeerJ. 2023 May 11;11:e15283. doi: 10.7717/peerj.15283 (PMC10183165; doi:10.7717/peerj.15283)
Supplement: Supplemental Information 5 [file peerj-11-15283-s005.docx]

**Table S5** Antimicrobial resistance (AMR) patterns and multiple antibiotic resistance (MAR) indexes of 36 *Vibrio parahaemolyticus* isolates

| **Isolate code** | **AMR pattern** | **MAR index** | |  |
| --- | --- | --- | --- | --- |
| VP 10/5 | AMP/COL | 0.074 |  | |
| VP 7 | AMP/COL | 0.074 |  | |
| VP 11 | AMP/COL/S | 0.11 |  | |
| VP 16 | AMP/COL/S | 0.11 |  | |
| VP 17 | AMP/COL | 0.074 |  | |
| VP 26 | AMP/COL/S | 0.11 |  | |
| VP 31 | COL | 0.04 |  | |
| VP 39 | AMP/COL/ FUR | 0.11 |  | |
| VP 41 | AMP/COL | 0.074 |  | |
| VP 42 | AMP/ CPM/ CTX/CAZ/CRO/FUR/COL/SIX | **0.3^#^** |  | |
| VP 46 | COL | 0.04 |  | |
| VP1/1 | AMP/COL/S | 0.11 |  | |
| VP 1/2 | AMP/COL | 0.074 |  | |
| VP 3/1 | AMP/COL/S | 0.11 |  | |
| VP 18/2 | AMP/COL | 0.074 |  | |
| VP 23/1 | COL | 0.04 |  | |
| VP 25/1 | AMP/COL | 0.074 |  | |
| VP 30/2 | AMP/COL | 0.074 |  | |
| VP 35/2 | AMP/COL | 0.074 |  | |
| VP 37/2 | AMP/COL | 0.074 |  | |
| SS4-002 | AMP/COL/SIX | 0.11 |  | |
| SS4-003 | AMP/COL/ FUR | 0.11 |  | |
| SS4-008 | AMP/COL | 0.074 |  | |
| SS4-009 | AMP/COL/S | 0.11 |  | |
| SS4-010 | AMP/COL | 0.074 |  | |
| SS4-012 | AMP/COL | 0.074 |  | |
| SS4-014 | AMP/COL | 0.074 |  | |
| SS4-016 | COL | 0.04 |  | |
| SS4-017 | COL | 0.04 |  | |
| SS4-082 | AMP/COL/SIX | 0.11 |  | |
| SS4-083 | AMP/COL | 0.074 |  | |
| SS4-084 | AMP/COL | 0.074 |  | |
| SS4-099 | AMP/COL | 0.074 |  | |
| SS4-179 | COL | 0.04 |  | |
| SS4-190 | AMP/COL | 0.074 |  | |
| SS4-218 | AMP/COL | 0.074 |  | |
|  |  |  |  | |

AMP, Ampicillin; CPM, Cefepime; CTX, Cefotaxime; CAZ, Ceftazidime, CRO, Ceftriaxone; FUR, Cefuroxime (sodium); COL, Colistin; S, Streptomycin and SIX, Sulfisoxazole.

^#^A bold letter indicates MAR index > 0.2.
