# Supplementary material for: Incidence, genetic diversity, and antimicrobial resistance profiles of Vibrio parahaemolyticus in seafood in Bangkok and eastern Thailand
Source: PeerJ. 2023 May 11;11:e15283. doi: 10.7717/peerj.15283 (PMC10183165; doi:10.7717/peerj.15283)
Supplement: Supplemental Information 6 [file peerj-11-15283-s006.docx]

**Table S6** Distribution of 25 sequence types (STs) with 12 known and 13 novel STs of 36 *Vibrio parahaemolyticus* isolated from seafood in Thailand

| **Isolate code** | ^#^**ST (%)** |
| --- | --- |
| SS4-008, SS4-009, SS4-010 | 197 (12%, 3/25) |
| VP25/1 | 212 (4%, 1/25) |
| SS4-002, SS4-003 | 413 (8%, 2/25) |
| VP10/5 | 813 (4%, 1/25) |
| SS4-099 | 818 (4%, 1/25) |
| VP37/2 | 1293 (4%, 1/25) |
| SS4-082, SS4-083, SS4-084, SS4-190, SS4-218 | 1925 (20%, 5/25) |
| SS4-016, SS4-017 | 2137 (8%, 2/25) |
| VP35/2 | 2195 (4%, 1/25) |
| VP30/2 | 2485 (4%, 1/25) |
| VP41 | 2621 (4%, 1/25) |
| VP23/1 | 2854 (4%, 1/25) |
| VP18/2 | **2916** (4%, 1/25) |
| VP16 | **2917** (4%, 1/25) |
| SS4-012 | **2918** (4%, 1/25) |
| VP3/1 | **2919** (4%, 1/25) |
| VP7, VP26 | **2920** (8%, 1/25) |
| VP1/2 | **2921** (4%, 1/25) |
| SS4-179 | **2922** (4%, 1/25) |
| VP42 | **2923** (4%, 1/25) |
| VP39 | **2924** (4%, 1/25) |
| VP1/1 | **2925** (4%, 1/25) |
| VP17, VP31, VP46 | **2926** (12%, 3/25) |
| SS4-014 | **2927** (4%, 1/25) |
| VP11 | **2928** (4%, 1/25) |
|  |  |

^#^Bold letters represent the novel STs.
